# Aerial Imagery Analysis – Quantifying Appearance and Number of Sorghum Heads for Applications in Breeding and Agronomy

**DOI:** 10.3389/fpls.2018.01544

**Published:** 2018-10-23

**Authors:** Wei Guo, Bangyou Zheng, Andries B. Potgieter, Julien Diot, Kakeru Watanabe, Koji Noshita, David R. Jordan, Xuemin Wang, James Watson, Seishi Ninomiya, Scott C. Chapman

**Affiliations:** ^1^International Field Phenomics Research Laboratory, Institute for Sustainable Agro-ecosystem Services, Graduate School of Agricultural and Life Sciences, The University of Tokyo, Tokyo, Japan; ^2^Agriculture and Food – Commonwealth Scientific and Industrial Research Organisation, St Lucia, QLD, Australia; ^3^Queensland Alliance for Agriculture and Food Innovation, The University of Queensland, Toowoomba, QLD, Australia; ^4^Montpellier SupAgro, Montpellier, France; ^5^Laboratory of Biometry and Bioinformatics, Department of Agricultural and Environmental Biology, Graduate School of Agricultural and Life Sciences, The University of Tokyo, Tokyo, Japan; ^6^Queensland Alliance for Agriculture and Food Innovation, The University of Queensland, Warwick, QLD, Australia; ^7^School of Agriculture and Food Sciences, The University of Queensland, Gatton, QLD, Australia

**Keywords:** high-throughput phenotyping, UAV remote sensing, sorghum head detecting and counting, breeding field, image analysis

## Abstract

Sorghum (*Sorghum bicolor* L. Moench) is a C4 tropical grass that plays an essential role in providing nutrition to humans and livestock, particularly in marginal rainfall environments. The timing of head development and the number of heads per unit area are key adaptation traits to consider in agronomy and breeding but are time consuming and labor intensive to measure. We propose a two-step machine-based image processing method to detect and count the number of heads from high-resolution images captured by unmanned aerial vehicles (UAVs) in a breeding trial. To demonstrate the performance of the proposed method, 52 images were manually labeled; the precision and recall of head detection were 0.87 and 0.98, respectively, and the coefficient of determination (*R*^2^) between the manual and new methods of counting was 0.84. To verify the utility of the method in breeding programs, a geolocation-based plot segmentation method was applied to pre-processed ortho-mosaic images to extract >1000 plots from original RGB images. Forty of these plots were randomly selected and labeled manually; the precision and recall of detection were 0.82 and 0.98, respectively, and the coefficient of determination between manual and algorithm counting was 0.56, with the major source of error being related to the morphology of plants resulting in heads being displayed both within and outside the plot in which the plants were sown, i.e., being allocated to a neighboring plot. Finally, the potential applications in yield estimation from UAV-based imagery from agronomy experiments and scouting of production fields are also discussed.

## Introduction

The grain yield of cereal crops is determined by accumulated processes of resource capture (e.g., radiation, water, and nutrients) that support net photosynthesis across the growing season (i.e., the carbohydrate source) and the utilization of this source, especially in the critical period around the reproductive stage which allows establishment of a potential sink (grain number) and, later in the crop, to fill those grains. These processes and their complex interrelationships form the basis of physiological models of crop growth and the development of crops such as sorghum (*Sorghum bicolor* L. Moench) (Hammer et al., 2010). Plant breeders and agronomists work collectively to modify these processes via genetics and management to develop cropping systems that optimize adaptation to different environments, particularly those associated with drought and heat (Lobell et al., 2015; Potgieter et al., 2016).

On an area basis, the final grain yield of cereal crops in a plot can be described as the product of average values of plant population, fertile head number per plant (i.e., main stem plus tillers), seeds per head, and individual seed mass. Insights into the changes in these component traits through the season and their final values at harvest provide researchers with a better understanding of crop adaptation, and potentially allow breeders to select for different combinations of these traits in different environments. The process of tillering provides a flexible or “plastic” response to challenging environments such as drought, and the trait of fertile head number per plant is under strong genetic control in both sorghum (Lafarge et al., 2002) and wheat (*Triticum aestivum*) (Mitchell et al., 2013; Dreccer et al., 2014). Although all of these component traits can be measured through labor-intensive hand-sampling methods, plant breeders and agronomists doing large trials will typically only use measures of yield (via plot harvester) and individual seed mass (via sample of grains from each plot). Together with estimates of plant population, which can be done by counting the emerged plants using ground and aerial images (Gnädinger and Schmidhalter, 2017; Jin et al., 2017; Liu et al., 2017), rapid and precise estimates of fertile head number per unit area would allow researchers to estimate the fertile head number per plant as an indicator of “tillering propensity.”

The aim of the research presented here was to develop a method that can detect and count the heads of sorghum from unmanned aerial vehicle (UAV) images, and then apply the method to specific plots to meet the needs of breeding programs. Machine-based image algorithms for detecting and counting an agriculture product with the use of harvesting robots and ground monitoring vehicles have been applied to imagery of grapes, tomato, apple, mango, and citrus fruits (Nuske et al., 2011; Payne et al., 2014; Sengupta et al., 2014; Yamamoto et al., 2014; Linker and Kelman, 2015; Gongal et al., 2016; Qureshi et al., 2016). However, these algorithms were designed to handle high-resolution images that do not include targets with large shape variations. Therefore, they are not suited for use with either the images or target object taken by UAVs in a breeding field of sorghum where different genotypes have heads that vary in color and shape, with these differences potentially changing with environment. In this paper, we propose a two-step machine-learning-based method that can detect and count sorghum heads from aerial images. To the best of our knowledge, this is the first report of research of this type.

## Materials and Methods

### Field Experiments and Image Acquisition

**FIGURE 1:**
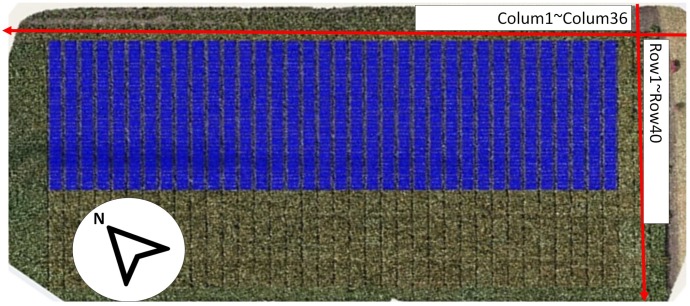
Experimental field layout.

The field experiments were part of multi-environment advanced yield-testing trials in a sorghum pre-breeding program. The trial was sown on 22 December 2015 at Hermitage, QLD, Australia (latitude: 28.21° S, longitude: 152.10° E, altitude: 459 m above sea level) during the 2015–2016 summer growing season. The target plant density was 115,000 plants/ha, with genotypes planted in plots comprising two 5-m-long rows. The plants were sown in plots within columns, and the trial used a solid row configuration, with a row spacing of 0.76 m between the two rows and a distance between two neighboring plots of 1 m as shown in Figure 1. In this trial, 1440 plots (laid out as 36 columns × 40 double-row plots; hereafter, we refer to double-row plots as rows, i.e., 36 columns × 40 rows) were sown, with several columns (216 plots in total) being “filler plots” to allow access for spraying. The trial comprised 22 check hybrids and 903 test-cross hybrids derived from crossing between a range of elite male parents and two female testers in the breeding program. The check hybrids were replicated at least four times, whereas 220 of the 903 test hybrids were replicated twice, with no replication at this site for the remaining 683 hybrids. The trial field was rain-fed and managed according to local management practices.

A UAV (Modified 3DR X8, Skywalker Technology Co., Ltd., China) was flown over the field with a pre-designed flight plan controlled with Mission Planner (open-source flight planning software for Pix Hawk autopilot^1^). The path included substantial overlap (i.e., 70% front-overlap and 80% side-overlap) at flight heights of 20 m and a flight speed of 3 m/s. The total flight time was approximately 50 min (five 10-min flights to cover the whole field). A commercial RGB camera (Sony Cyber-shot DSC-RX100M3, Tokyo, Japan) was mounted on the UAV in a landscape format. The resolution of the camera was set to 5472 × 3648 pixels, which resulted in an average ground sampling distance of 0.45 cm at 20 m height with a footprint of 20 m. The image sets were captured at 1-s intervals during the flights, so that about 2000 images were produced for the target field (about 35 GB+) per flight.

### Data Preparation

**FIGURE 2:**
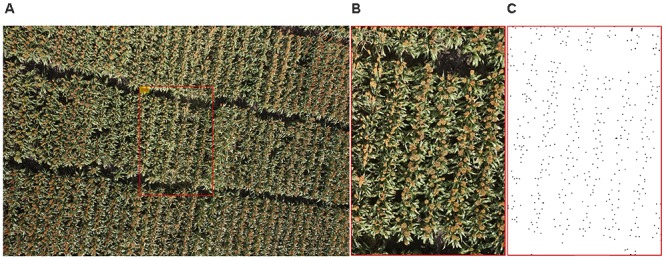
An example of image preparation for algorithm development: **(A)** original image, **(B)** cropped image, and **(C)** manually labeled cropped image, the points represent the heads (dataset 1).

Flight data obtained on 24 March 2016 were chosen for this study because almost all of the genotypes were heading stages at this time, so that the widest diversity of heads in terms of color and shape could be found in these images and the dataset would be large and balanced enough for later processing. We believe, however, that the image processing algorithm conducted on this dataset has the general capabilities to be used on the whole dataset, including images taken at different times of the growth season. From the 2109 original images obtained on 24 March 2016 when most of the plants in the experiment had produced heads (on average, this date was about one to 2 weeks after anthesis), 52 images were randomly selected following an uniform distribution to develop and test the head detecting and counting algorithm. To minimize the influence of camera lens distortion, all of the images were cropped so that about 10% of image was used (Figure 2). The original image of 5472 × 3648 pixels was cropped to a central region of 1154 × 1731 pixels, which corresponded to an area of 2.3 m × 3.5 m which contained three to five plots. All of 52 cropped images were carefully hand labeled with points in Adobe Photoshop (Adobe Systems Inc., San Jose, CA, United States) as shown in Figure 2C. These images were grouped as Dataset 1.

### Sorghum Head Detection

**FIGURE 3:**
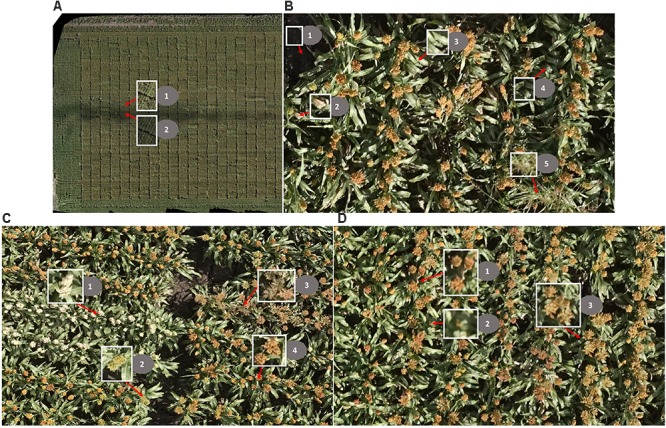
Challenges of head detection in a real field. **(A)** Changing light conditions within one flight: (1) images taken under sunny conditions; (2) images taken under cloudy conditions. **(B)** Complex background: (1) soil/ground (shadowed partially/fully), (2) dead leaves, (3) green leaves, (4) shadowed leaves, and (5) grass. **(C)** The sorghum heads vary in color: (1) white, (2) green, (3) brown, and (4) orange. **(D)** The sorghum heads vary in size and shape: (1) heads from main stem, (2) heads from tillers, and (3) overlapping heads; note that the shape of the heads is compact in 1 and 2 but is expanded in 3.

The main challenges of creating an image-based solution in a real breeding field are: (1) changing light conditions within a single flight (images vary in color; Figure 3A); (2) complex background (Figure 3B); and (3) head variations in color, size, and shape caused by light conditions, genotype, heading stage, source of head (main stem or tillers), angle of head stands, and overlapping of heads (Figures 3C,D).

To overcome the first challenge, in our previous work (Potgieter et al., 2015; Guo et al., 2016), we proposed a two-step machine-learning, voting-based method. The method uses colors (RGB, HSV [hue, saturation, and value], Lab from related color space, ExG [excess green], and ExR [excess red]) introduced by Meyer and Neto (2008); texture features (average gray level, average contrast, measure of smoothness, third moment, measure of uniformity, and entropy) from gray-scale imagery introduced by Gonzalez et al. (2010); and contrast, correlation, energy, and homogeneity from the gray-level co-occurrence matrix introduced by Haralick et al. (1973) to train several decision-tree-based pixel segmentation models (DTSM) (Guo et al., 2013). These DTSM models are then used to segment the images to sorghum and non-sorghum head regions. Based on the segmented regions created in the last step, a bag of visual words approach, modified from Guo et al. (2015), is applied to the test images again to gain a new segmentation image with misclassifications removed from the previous step. Finally, a voting process is used for all the segmented images to acquire the most reliably detected region of the sorghum heads. With only 20 test images cropped from a GoPro^TM^ Hero4 camera (GoPro, Inc., San Mateo, CA, United States), the method showed good accuracy for sorghum head detection; the precision (the proportion of correctly detected head region inside true head region) and recall (the proportion of correctly detected head region inside detected head region) were 0.95 and 0.96, respectively (Guo et al., 2016). However, since this method used texture feature and sliding window, the computation time and cost was substantial for processing of high resolution images and did not suit practical use.

**FIGURE 4:**
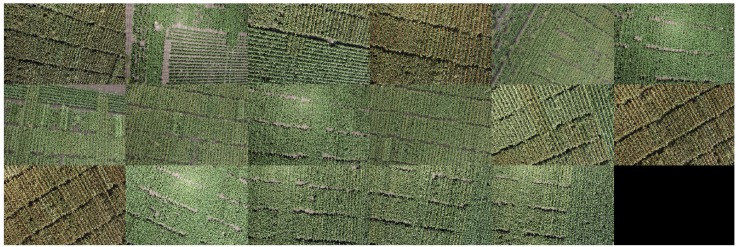
Dataset 0 comprised 17 images for training data collection of pixel-based segmentation model. The images were selected considering light condition, head color, head shape, and background.

**FIGURE 5:**
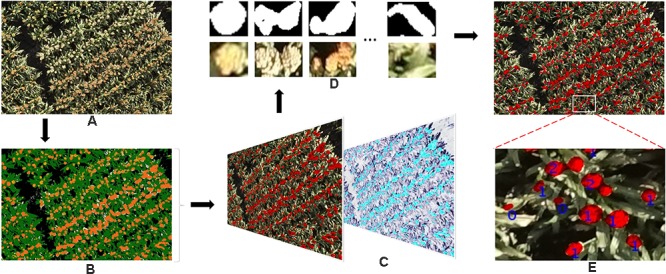
The work flow of the proposed method of detecting and counting sorghum heads. **(A)** Original image. **(B)** Pseudo-color image demonstrating pixel classification result by DTSM: white head, yellow; soil, gray; shadows, black; dead leaves, off-white; leaves, green; orange heads, dark orange; and green heads, light orange. **(C)** Detected head regions (left) and overlapped with manually pointed head image (right). The black dots indicate heads pointed manually with Photoshop. **(D)** The head regions cropped from original images based on **(C)**. **(E)** Detected head regions and number of heads counted. The numbers shown in the image indicate the number of the sorghum heads; 0 means incorrect detection..

Using the knowledge gained from our previous studies (Potgieter et al., 2015; Guo et al., 2016), here we only used color features to train a pixel-based segmentation model. First, seven classes – (1) background soil, (2) background shadow, (3) background dead leaves, (4) leaves, (5) green heads, (6) orange heads, and (7) white heads – were defined. For each class, a series of nine color features (r, g, b; H, S, V; L^∗^, a^∗^, and b^∗^) from three standard color spaces were carefully collected from 17 images (Figure 4, Dataset 0) that were selected from the entire image dataset of 2109 images, considering the diversity of lighting conditions and head colors. Using these features, we trained a DTSM model and applied it to all of the test images to classify their pixels into the seven classes. DTSM is a supervised machine learning approach based on the decision tree (DT) (Guo et al., 2013, 2017). This approach generates a decision tree model using the selected color features and corresponded classes, then a constructed tree model is applied to segment test images, such that each pixel becomes assigned to one of the classes (Figures 5A,B). After this, the head-related pixels (green heads, orange heads, and white heads) were selected and integrated together into “head regions,” as shown in Figure 5C.

### Sorghum Head Counting

To count the number of detected regions (Figure 5D) from the first step with a reliable model, we randomly separated Dataset 1 into six sets, each set being eight or nine images. The images from five of the sets were used to train the model with fivefold cross validation, and the last set was used to estimate the performance of the model. In detail, hand-labeled images were used to extract the 11 morphology features of all of the candidate head regions and the corresponding head numbers (Figures 5C,D):

(1) Area: actual number of pixels in each candidate head region.
(2) Eccentricity: eccentricity of the ellipse that has the same second-moments as the candidate head region.
(3) Extent: ratio of pixels in the candidate head region to pixels in the total bounding box.
(4) Perimeter: total number of pixels around the boundary of the candidate head region.
(5) Major axis length: length of the major axis of the ellipse that has the same normalized second central moments as the candidate head region.
(6) Minor axis length, length of the minor axis of the ellipse that has the same normalized second central moments as the candidate head region.
(7) ConvexArea, number of pixels in smallest convex polygon that can contain the candidate head region.
(8) FilledArea: number of pixels in each candidate head region with all holes filled in.
(9) EquivDiameter: diameter of a circle with the same area as the candidate head region.
(10) Solidity: proportion of the pixels in the convex hull that are also in the candidate head region.
(11) Roundness: circularity of candidate head region.

These features of each candidate head region were then used as predictors with corresponded head numbers as the response, in order to train a Quadratic-SVM (Support Vector Machine) classifier with fivefold cross validation. Support Vector Machine is a supervised machine learning algorithm which has become commonly used to solve classification problems. SVMs are based on the idea of finding a hyperplane that best divides a dataset into two classes. In this paper, a quadratic kernel is used, as it is less computationally intensive but has been show to perform as well as previous work (Guo et al., 2016).

Then model was applied to all of the candidate regions from step 1 to count the numbers of heads in each image (Figure 5E). The training data and a guidance is also provided in Supplementary Materials, which can support opportunities for readers to test other classifiers (such as Decision Trees, Random Forest, SVMs with different kernel functions) using the MATLAB “*Classification Learner*” application.

### Application of Method to Count Heads Within Individual Plots

**FIGURE 6:**
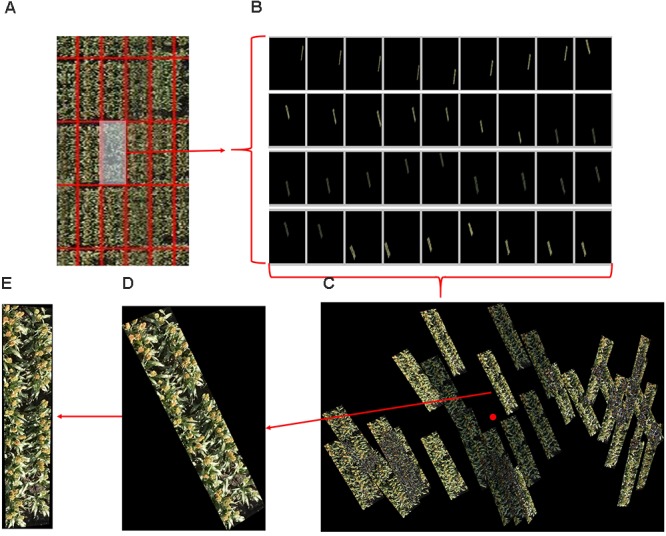
An example of plot segmentation and identification from original images. **(A)** A plot is selected from a set of ortho-mosaic images. **(B)** The selected plot appears in several original images but in different locations. **(C)** The plots images are grouped and one is selected based on its distance from the central part of the image. **(D)** The selected plot is cropped from the corresponding original image. **(E)** The selected plot is rotated based the corner detection and orientation calculations of **(D)**.

In total, 2109 original images were also processed by the Pix4Dmapper software package (Pix4D, SA, Lausanne, Switzerland) to generate 3D point cloud and ortho-mosaic images of the whole experimental field. The ortho-mosaic images were segmented into individual plots and projected back to the corresponding original images to segment the original pixels (cf. mosaic) following our previously reported method (Duan et al., 2016). In total, 28,825 individual plot images (i.e., many replications of each of the 1440 plots) were segmented from the dataset. The process of plot segmentation and identification from the original images is shown in Figure 6. Any given plot can appear in several original images but in different locations (Figures 6A,B); the plot with the shortest Euclidean distance to the central part of the image was selected as the candidate plot image and cropped from the original image and rotated with calculated orientation (Figures 6C,D). Each plot was thereby generated from 1440 images, and 40 of them were randomly selected for this study. To validate the accuracy of the proposed detection and counting method, each plot image was also carefully hand labeled by two scientists (Dataset 2).

## Results and Discussion

Datasets 1 and 2 were both used to evaluate the head detecting and counting capabilities of the proposed method.

**FIGURE 7:**
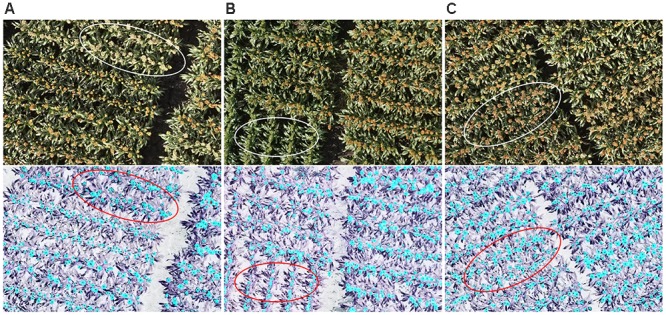
An example of head detection. Images contain **(A)** white heads, **(B)** green heads, and **(C)** brown heads. All of the images contain orange heads. The upper panels show the original images and the lower ones show the detected head regions (blue) and hand-labeled head centers (black dots). Almost all of the heads of the different colors were detected by the proposed model.

Figure 7 shows head detection results from dataset 1. Almost all of the heads of different colors, shapes, and sizes were successfully detected. Table 1 presents an evaluation of the detection results in terms of precision and recall based on the definitions of Davis and Goadrich (2006). Precision indicates that for the total number of head regions, which proportion were correctly detected (with a ratio of 1.0 being perfect) while recall indicates for all detected regions, how many are correctly detected (perfect = 1.0). The algorithm was able to accurately detect 87% of sorghum heads for dataset 1 and 82% for dataset 2, and the accuracy rates were high (recall = 0.98) for both datasets.

**Table 1:** Evaluation of the detection results.

Dataset	TP	FP	FN	Precision	Recall	*F*-measure
1 (52 images)	15,773	2434	314	0.87	0.98	0.92
2 (40 plots)	2762	587	44	0.82	0.98	0.89


*Images from datasets (1 and 2) had not been used to train the detection model.*

*TP (True Positive): head region correctly detected.*

*FP (False Positive): non-head region erroneously detected as a head.*

*FN (False Negative): head region undetected.*

*Precision = TP/(TP + FP). Recall = TP/(TP + FN).*

*F-measure = (2 × precision × recall)/(precision + recall).*

**FIGURE 8:**
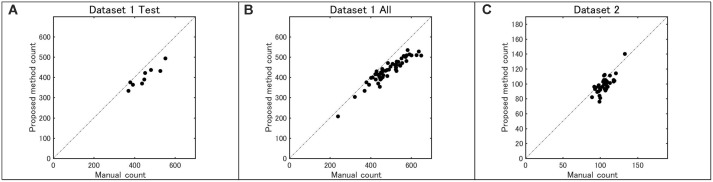
Accuracy of head number determined by the proposed method as compared with that done by manual counting: **(A)** dataset 1 test, **(B)** dataset 1 (all), and **(C)** dataset 2 (all).

Figure 8 shows the counting accuracy of the proposed method. First, the total number of sorghum heads in each image from both datasets was counted and double checked carefully by two researchers. Then, the test part of dataset 1, all of dataset 1, and all of dataset 2 were tested by the model, and the coefficients of determination (*R*^2^) were 0.85, 0.88, and 0.56, respectively.

**FIGURE 9:**
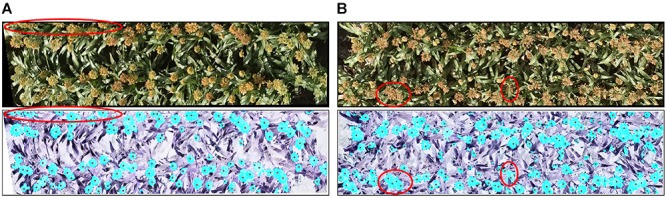
Reasons for incorrect counting: **(A)** the plot segmentation was not perfect, so parts of some heads were cut out (upper red oval); **(B)** some regions included multiple overlapping heads (left red circle), and some heads were covered by leaves (right red circle). The upper panels show the original images and the lower ones show the detected head regions (blue) and hand-labeled head centers (black dots).

The *R*^2^ of the head counting at the image level (Dataset 1) was relatively high, however, it was observed to be decreased substantially when applying the model to single plot images (Dataset 2). The main reason for this is likely related to the application of the plot segmentation algorithm in this experiment. In our case, there were only two rows in each plot, and hence it was common to cut out parts of the heads (which overlapped “plot” boundaries) during segmentation (Figure 9A). Plot segmentation accuracy could be improved either by redesigning the field experiment to enlarge plot size or by using drones with a better positioning system and a higher-resolution camera. Alternatively, we could try applying methods to the rows of heads in a multi-plot image to try to better delineate the boundaries between the plots (i.e., tracing around the heads at edges of plot).

For both of the datasets, challenges remain in dealing with the large variability among genotypes, growth stages, and growth position (main stem or tillers) of heads, all of which contribute to large differences in the morphological features of detected head regions. As shown in Figure 9B, detected regions may comprise multiple overlapping heads while other heads may be obscured by leaves, neither feature of which has been trained in the counting model. The model capabilities could therefore be improved with additional training data and exploring more efficient features.

With the rapid development of GPU technology in recent years, the size of electrical infrastructure has been decreased significantly without loss in performance. These types of embedded platforms allow for onboard real-time image processing, so that the proposed or other methods could be applied with real-time image input. By integrating such system with UAVs, scouting of production fields could be completed by the end of the flight.

## Conclusion

We proposed a simple two-step machine-learning-based image processing method to detect and count the number of sorghum heads from high-resolution images captured by UAVs in a breeding field. This introduces realistic challenges given that sorghum has various genotypes with different growth stages, and the heads can have different colors, shapes, and sizes. Using carefully selected training data, the precision and recall of head detection were 0.87 and 0.98, respectively, for dataset 1 and 0.82 and 0.98 for dataset 2. The coefficients of determination (*R*^2^) for head counting were 0.88 and 0.56 for datasets 1 and 2, respectively.

Head number per unit area is an important component of the yield of cereal crops. As well as being useful to agronomists and breeders, the method described here has utility in production agriculture, e.g., by using UAVs to survey a field to estimate head number, and then manually sampling a range of head sizes in order to estimate yield as product of weighted average head size (grain weight per head) and head number. Counting can also be used to characterize spatial variability in the field, as well as non-uniformity of development or head size over time (multiple monitoring flights). In a research context, the ability of this method to count heads of contrasting genotypes in diverse measurement conditions provides a better capability to estimate head number in plant breeding trials. The main limitation at present in being able to correctly delineate the boundaries of plots and we are investigating ways of doing this, e.g., by defining plots early in the season and tracking the head positions relative to original plant positions.

The application of machine-learning-based image analysis technologies is become increasingly important in field-based plant phenotyping tasks. By using these rapidly improving techniques, we believe the accuracy of phenotyping will increase while the computational cost will decrease, both of which will help researchers reach the goal of real-time phenotyping (Fuentes et al., 2017; Naik et al., 2017). However, key techniques such as training data preparation, model selection, and feature definition still rely on highly specialized knowledge in both plant science and computer science (Singh et al., 2016). Deep learning is a possible solution to reduce the difficulties, but generating ground-truth data (image annotation) to train the models is still very labor intensive (Sa et al., 2016, 2017; Ghosal et al., 2018). A method is needed to automatically generate reliable training data, and the detection feature of the proposed method could be used as a semi-automatic tool to provide candidate training datasets. We encourage the plant research community to share the existing annotated dataset to accelerate plant-phenotyping community growth in a similar way that ImageNet is used (Russakovsky et al., 2015). To aid in this growth, datasets 1 and 2 along with the manual labeling used in this study are available in the Supplementary Materials.

## Author Contributions

WG analyzed the data and interpreted results with the input of JD. AP, BZ, SC, KW, KN, and SN conceived the research. WG and XW labeled the images. DJ and XW conceived, designed, and coordinated the field experiments. WG wrote the paper with input from all authors.

## Conflict of Interest Statement

The authors declare that the research was conducted in the absence of any commercial or financial relationships that could be construed as a potential conflict of interest.

## Footnotes

1 http://planner.ardupilot.com/
