# Supplementary material for: Aerial Imagery Analysis – Quantifying Appearance and Number of Sorghum Heads for Applications in Breeding and Agronomy
Source: Front Plant Sci. 2018 Oct 23;9:1544. doi: 10.3389/fpls.2018.01544 (PMC6206408; doi:10.3389/fpls.2018.01544)

Supplementary Materials

Note: all the original files could be found from our support page:

<https://github.com/oceam/sorghum-head>

1. Supplementary Materials 1:
2. Original image dataset 0, 17 training images for color feature selection.
3. DTSM_trainingData.txt, Selected color features in CSV format for generate DTSM model
4. Supplementary Materials 2:
   1. featureTable_croped_img_rand_201808.csv, Selected 11 morphologic features in CSV format for generate SVM model.
   2. Guide of comparing different machine learning models in MATLAB.
      1. Save featureTable_croped_img_rand_201808.csv to excel or import it into Matlab work space.
      2. Open Matlab “*Classification Learner APP*”.
      3. Import data from 1), define 11 morphologic as “predictor” and CountHeads as “response”, then choose cross-validation method.
      4. follow the steps introduced by Matlab to train and compare different machine learning algorithms.

“https://jp.mathworks.com/products/statistics/classification-learner.html”

1. Supplementary Materials 3:
   1. original image dataset 1 and corresponded Dot labeled data (52 cropped images).
   2. original image dataset 2 and corresponded Dot labeled data (40 plot images).


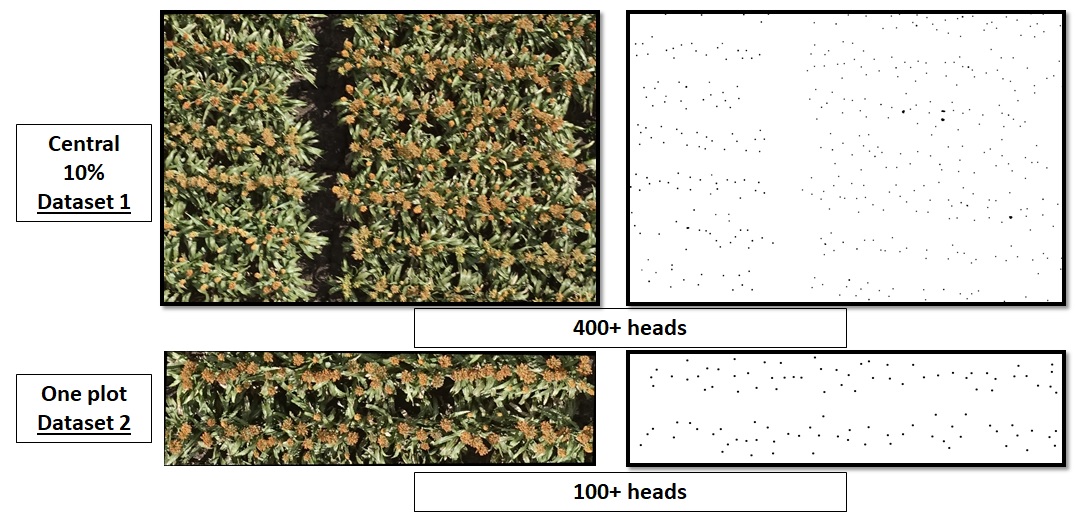


1. Supplementary Materials 4: Flowchart of whole processing.


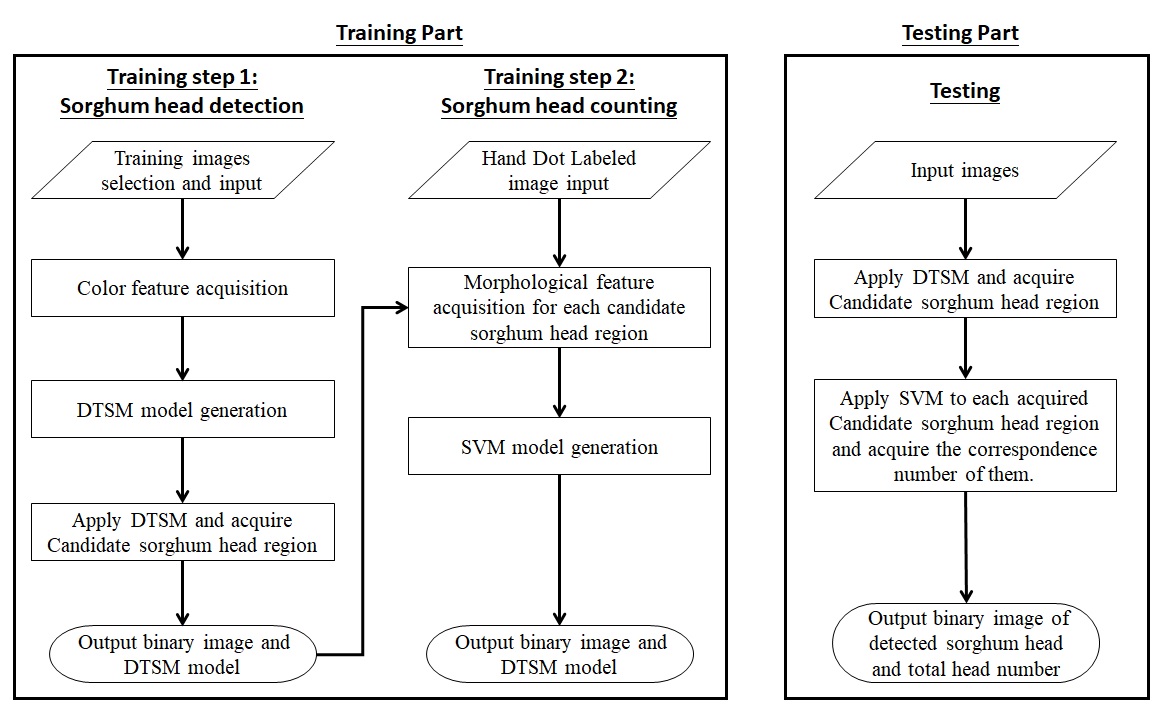

Supplement: Supplementary file 1 [file Table_1.DOCX]
